# Retrospective Analysis of the Effectiveness of Oral Semaglutide in Type 2 Diabetes Mellitus and Its Effect on Cardiometabolic Parameters in Japanese Clinical Settings

**DOI:** 10.3390/jcdd10040176

**Published:** 2023-04-18

**Authors:** Hodaka Yamada, Masashi Yoshida, Shunsuke Funazaki, Jun Morimoto, Shiori Tonezawa, Asuka Takahashi, Shuichi Nagashima, Kimura Masahiko, Otsuka Kiyoshi, Kazuo Hara

**Affiliations:** 1 Department of Medicine, Division of Endocrinology and Metabolism, Jichi Medical University Saitama Medical Center, 1-847 Amanuma-cho, Omiya-ku, Saitama 330-8503, Japan; 2 Department of Pharmacy, Jichi Medical University Saitama Medical Center, 1-847 Amanuma-cho, Omiya-ku, Saitama 330-8503, Japan

**Keywords:** glucagon-like peptide-1 receptor agonist, oral semaglutide, type 2 diabetes mellitus, real-world data, cardiometabolic risk factors

## Abstract

Glucagon-like peptide-1 receptor agonists (GLP-1RA) have a more potent glycated hemoglobin (HbA1c)-lowering effect than existing therapies and are widely used for treating type 2 diabetes mellitus (T2DM). Once-daily oral semaglutide is the world’s first oral GLP-1RA. This study aimed to provide real-world data on oral semaglutide in Japanese patients with T2DM and its effects on cardiometabolic parameters. This was a single-center retrospective observational study. We examined changes in HbA1c and body weight (BW) and the rate of achieving HbA1c < 7% after 6 months of oral semaglutide treatment in Japanese patients with T2DM. Furthermore, we examined differences in the efficacy of oral semaglutide with multiple patient backgrounds. A total of 88 patients were included in this study. Overall, the mean (standard error of the mean) HbA1c at 6 months decreased by −1.24% (0.20%) from baseline, and BW at 6 months (*n* = 85) also decreased by −1.44 kg (0.26 kg) from baseline. The percentage of patients who achieved HbA1c < 7% changed significantly from 14% at baseline to 48%. HbA1c decreased from baseline regardless of age, sex, body mass index, chronic kidney disease, or diabetes duration. Additionally, alanine aminotransferase, total cholesterol, triglyceride, and non-high-density lipoprotein cholesterol were significantly reduced from baseline. Oral semaglutide may be an effective option for the intensification of therapy in Japanese patients with T2DM who have inadequate glycemic control with existing therapy. It may also reduce BW and improve cardiometabolic parameters.

## 1. Introduction

Glucagon-like peptide-1 receptor agonists (GLP-1RA) are widely used for treating type 2 diabetes mellitus (T2DM) as they lower blood glucose more potently than conventional oral drugs and reduce body weight (BW) with less risk of hypoglycemia [1,2,3,4]. Existing GLP-1RA could only be administered by injection, but once-daily oral semaglutide was developed, which is absorbed through the stomach when co-administered with an absorption enhancer, sodium N-[8-(2-hydroxybenzoyl)amino] caprylate) [5]. Multiple Peptide Innovation for Early Diabetes Treatment (PIONEER) trials, including PIONEER 6 as a cardiovascular outcome trial, showed that oral semaglutide is safe and effective [6,7,8]. Oral semaglutide can be a promising and powerful treatment option for patients who wish to intensify their treatment with oral medications [9]. A systematic review and meta-analysis of GLP-1RA showed beneficial effects on cardiovascular (major adverse cardiovascular event (MACE)), mortality, and kidney outcomes in patients with T2DM [10]. However, the details of the MACE benefit of GLP-1RA are not yet clear, but it is assumed to be mediated by lowering blood pressure, improving atherosclerosis-induced lipid profiles, and acting on the vascular endothelium [11,12]. Additionally, a recent systematic review showed that Asian patients with T2DM might derive greater MACE benefits from GLP-1R than Whites [13]. From the above, it is necessary to understand the background of the trials conducted in Japan, the US, and Europe. A post hoc analysis of PIONEER 9 and PIONEER 10 in Japanese T2DM was recently reported and found to be effective across a range of baseline characteristics [14]. Along with the above randomized controlled trials (RCTs), real-world data (RWD) on Japanese patients with T2DM are considered important for more practical diabetes treatment decisions. Considering the differences in the pathogenesis of basic T2DM, unlike Caucasians, whose condition of T2DM is characterized by obesity and insulin resistance, East Asians, including the Japanese, are characterized by an early decline in insulin secretory capacity, especially the first phase of insulin [15,16]. In particular, GLP-1RA enhances the first phase of insulin secretion [17,18]. Several RCTs pointed out that the HbA1c-lowering effect of GLP-1RA, including semaglutide, may be greater in Asian populations, including the Japanese, than in Caucasian-centered global populations, and the pathophysiology of insulin deficiency described above is thought to be involved [14,15,16,19,20,21]. Oral semaglutide (3 mg, 7 mg, and 14 mg) became available in Japan in February 2021. However, there are few reports of RWD in Japanese patients with T2DM. In fact, oral GLP-1RA has the advantage of being more easily administered to a larger number of patients with diabetes than injectable formulations [9], and RWD on effectiveness and safety analyzed in a wide range of patients would be useful. This study aimed to collect short-term RWD on oral semaglutide effectiveness, safety, and impact on cardiometabolic parameters in a real-world clinical setting in Japan.

## 2. Materials and Methods

### 2.1. Study Design and Population

In a previous retrospective study, we examined the effects of once-weekly semaglutide [22]. This study was conducted using essentially the same design [22]. The detailed methodology has been described below. This was a retrospective cohort study conducted at a single center. The study included patients with T2DM treated with oral semaglutide at the Jichi Medical University Saitama Medical Center between January 2022 and July 2022. In all patients, Oral semaglutide was initiated in accordance with the package insert instructions for intensifying diabetes treatment. Specifically, once daily administration of 3 mg oral semaglutide was initiated for the patient, and the dose was increased to 7 mg once daily after 4 or more weeks. If the effect was insufficient, the dose was increased to 14 mg once daily at the physician’s discretion. We evaluated clinical parameters 6 months after oral semaglutide initiation. The inclusion criteria included Japanese patients with T2DM who initiated oral semaglutide in an outpatient setting. The exclusion criteria included patients with acute metabolic disorders, such as diabetic ketoacidosis and hyperosmolar hyperglycemic syndrome, those who were taking steroids, had an acute infectious disease or had any newly diagnosed cancer, those who required hospitalization for another disease treatment, those who exhibited a change in an oral anti-diabetic drug (OAD) or new insulin induction during the observation period, and those whose adverse events led to discontinuation within 6 months.

**Figure 1:**
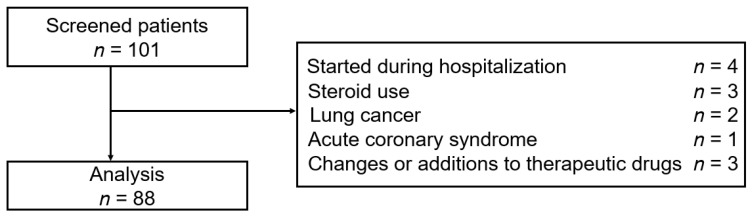
Flowchart of patient enrollment.

Figure 1 shows the flowchart of patient enrollment. A total of 101 patients were selected for screening. After applying the inclusion and exclusion criteria, we finally analyzed the data from 88 patients (full analysis set).

The primary endpoint was the change in HbA1c and BW from baseline to 6 months after the initiation of oral semaglutide. The secondary endpoint was the proportion of participants achieving HbA1c < 7.0%. As an exploratory endpoint, we evaluated baseline characteristics involved in HbA1c change, effects of different semaglutide doses (3–14 mg) on changes in HbA1c and BW, effects on cardiometabolic parameters, and the information of adverse events. We obtained baseline demographic data of the patients, including age, sex, body mass index (BMI), diabetes duration, presence of macroangiopathy and cardiovascular disease, concomitant medications, and adverse events, from medical records. Additionally, we collected metabolic parameters (non-fasting state), including HbA1c, total cholesterol (TC), high-density lipoprotein cholesterol (HDL-C), triglyceride (TG), non-HDL-C, aspartate aminotransferase (AST), alanine aminotransferase (ALT), and estimated glomerular filtration rate (eGFR). We calculated non-HDL-C using the following equation: TC−HDL-C (non-fasting state). This study was conducted in accordance with the Declaration of Helsinki of 1964, as revised in 2013, and approved by the Ethics Committee of Jichi Medical University Saitama Medical Center (No. S19-005).

### 2.2. Statistical Analysis

Continuous variables were expressed as means (±standard error of the mean, SEM) or medians with interquartile range (IQR), and categorical variables as numbers or percentages. Each parameter was compared before and after oral semaglutide initiation using the Wilcoxon signed-rank sum test. The clinical characteristics were compared using the Kruskal–Wallis test among three groups. Categorical variables were compared using McNemar’s test. Patients who showed a change in HbA1c greater than 1.0% were defined as the good response group. Multivariate logistic regression analysis was performed to explore the effects of various potential factors on the HbA1c reduction of oral semaglutide in the good response group.

The patients were divided into the following groups: the younger and elderly groups (age, <65 and ≥65 years), the non-obese and obese groups (BMI, <25 and ≥25 kg/m^2^), and the chronic kidney disease (CKD) and non-CKD groups (eGFR, <60 and ≥60 mL/min/1.73 m^2^). The baseline HbA1c was divided into <8.5% or ≥8.5%, and the diabetes duration was divided into <10 or ≥10 years based on these mean values. All statistical analyses were performed using EZR (Jichi Medical University, Saitama Medical Center), a graphical user interface for R (The R Foundation for Statistical Computing), and a modified version of R commander designed to add statistical functions frequently used in biostatics [23]. A *p* value < 0.05 indicated statistical significance.

## 3. Results

### 3.1. Demographics

Table 1 shows the baseline characteristics of the 88 patients. Weight data were available for 85 patients at 6 months. The median (IQR) age at baseline was 62 (53.8–68) years, with 62.5% (*n* = 55) being males. The baseline mean (SEM) HbA1c and BMI were 8.53 (0.17) % and 27.3 (0.61) kg/m^2^, respectively. The elderly group (age, ≥65 years) included 32 patients, and the obese group (BMI ≥ 25 kg/m^2^) included 59 patients. The majority of patients (54.6%) were treated with OADs only, and the most used OADs were the sodium-glucose transport protein 2 inhibitors (68.2%), dipeptidyl peptidase-4 inhibitors (46.6%), and biguanides (44.3%). One-third of the patients (35.2%) used injectable GLP-1RA. GLP-1RA was most commonly administered as a weekly injection of dulaglutide in 17 patients (0.75 mg in all patients) and once-weekly semaglutide in 8 patients (0.25 mg in 1 and 0.5 mg in 7 patients). In all patients, oral semaglutide was initiated at 3 mg. After 6 months, the semaglutide doses were 3 mg in 13 patients, 7 mg in 53 patients, and 14 mg in 22 patients (mean, 8.16 mg). There were 70 patients (79.5%) who were attached to some kind of job and 22 patients (25%) with a documented regular exercise routine.

### 3.2. Changes in HbA1c and BW

**Figure 2:**
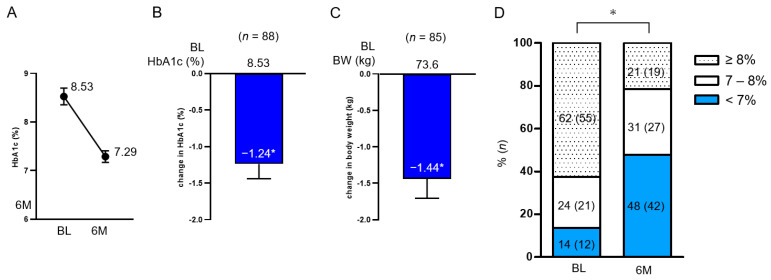
Change in HbA1c (**A**,**B**) and BW (**C**) from baseline overall at 6 months and the proportion of patients achieving HbA1c < 7% (**D**). Data in the figure are shown as mean with SEM. * *p* < 0.001 in the Wilcoxon signed-rank sum test (**B**,**C**) and McNemar’s test (**D**) (vs. BL). BL, baseline; HbA1c, glycated hemoglobin; BW, body weight; SEM, standard error of the mean.

**Figure 3:**
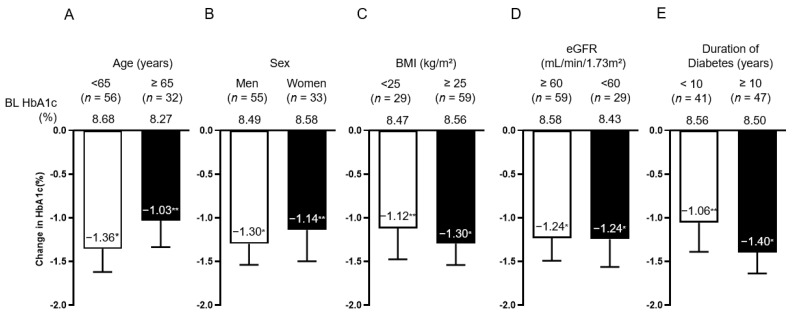
Change in HbA1c by subgroup; age (**A**), sex (**B**), BMI (**C**), eGFR (**D**), and diabetes duration (**E**). Data in the figure are presented as mean with SEM. * *p* < 0.001; ** *p* < 0.01 in the Wilcoxon signed-rank sum test (vs. BL HbA1c). BL, baseline; HbA1c, glycated hemoglobin; BMI, body mass index; eGFR, estimated glomerular filtration rate; SEM, standard error of the mean.

Overall, the mean (SEM) reduction in HbA1c at 6 months was −1.24% (0.20%) from baseline, and BW at 6 months was also reduced by −1.44 kg (0.26 kg) from baseline (Figure 2A–C). The percentage of patients who achieved HbA1c < 7% significantly changed from 14% at baseline to 48% (Figure 2D). The degree of change in HbA1c between the two groups divided in the methods section did not differ by age, sex, BMI, eGFR, and diabetes duration, and in both groups, HbA1c was significantly reduced from baseline (Figure 3). BMI (<25, <30, and ≥30 kg/m^2^) and diabetes duration (<10, <20, and ≥20) were further examined in three subgroups, and HbA1c was significantly reduced from baseline (Figure S1 in the Supplementary Materials).

Multivariate analysis showed that the improvement in HbA1c at 6 months (the good response) was greater when baseline HbA1c was higher (Table S1 in the Supplementary Materials). The effect of oral semaglutide on lowering HbA1c was greater in the group with baseline HbA1c of 9% or higher than that in the group with baseline HbA1c of less than 8% (Figure S2 in the Supplementary Materials).

**Figure 4:**
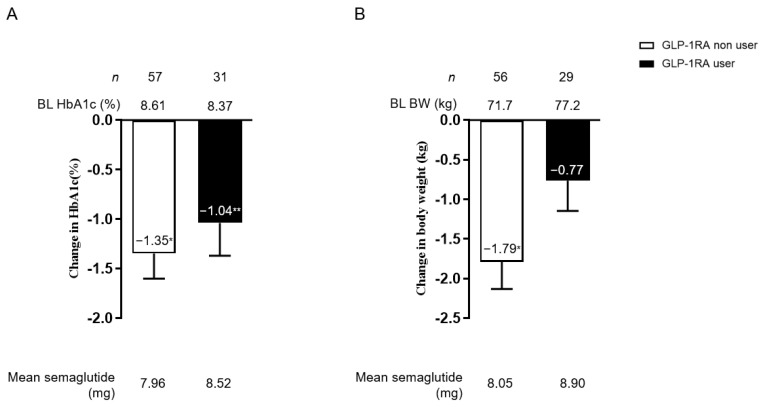
Change in HbA1c (**A**) and BW (**B**) from baseline among prior GLP-1RA non-users and users. White bars indicate GLP-1RA non-users and black bars indicate GLP-1RA users. The mean oral semaglutide dosage is shown at the bottom of the figure. Data in the figure are presented as mean with SEM. * *p* < 0.001; ** *p* < 0.01 in the Wilcoxon signed-rank sum test (vs. BL HbA1c). BL, baseline; HbA1c, glycated hemoglobin; BMI, body mass index; GLP-1RA, glucagon-like peptide-1 receptor agonist; SEM, standard error of the mean.

HbA1c and weight change from baseline were examined in the prior GLP-1RA user and non-user subgroup after oral semaglutide induction. The HbA1c was improved significantly from baseline in both groups (no difference between the two groups, *p* = 0.490). The BW change showed significant change in the prior GLP-1RA non-user group. The degree of BW change between the two subgroups was significantly different (*p* = 0.025) (Figure 4). The mean dose of oral semaglutide was not different between the two subgroups (prior GLP-1RA user or not) (*p* = 0.512 in Figure 4A and *p* = 0.281 in Figure 4B). In the analysis involving OAD use prior to the introduction of oral semaglutide, both HbA1c and body weight decreased from baseline, regardless of whether dipeptidyl peptidase-4 inhibitors were used or whether the patient was treated only with OAD (Figure S3 in the Supplementary Materials).

**Figure 5:**
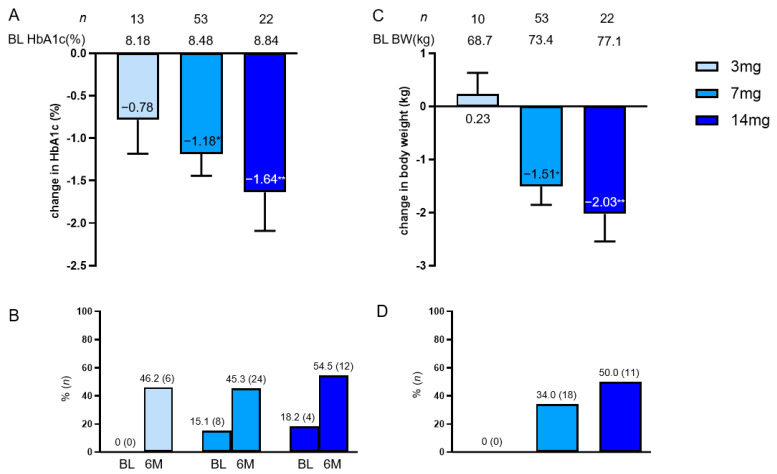
Change in HbA1c (**A**) and BW (**C**) per oral dose of semaglutide (3, 7, and 14 mg) and the percentage of patients who achieved HbA1c < 7% (**B**) and weight loss of 3% or greater (**D**) at 6 months. Data in the figure are presented as mean with SEM or number (%). * *p* < 0.001; ** *p* < 0.01 in the Wilcoxon signed-rank sum test (vs. BL HbA1c). BL, baseline; HbA1c, glycated hemoglobin; BW, body weight; SEM, standard error of the mean.

Semaglutide dose-dependently increased the mean HbA1c decrease from baseline and the rate of achieving HbA1c < 7%. However, no statistically significant differences were observed among the three groups (*p* = 0.665) (Figure 5A,B). Furthermore, no difference in BW change was observed among the three groups (*p* = 0.096), and the overall weight loss percentage from baseline of 3% or more was 34.1%, reaching 50% in the semaglutide 14 mg group (Figure 5C,D).

### 3.3. Evaluating Cardiometabolic Parameters

We evaluated oral semaglutide effects on cardiometabolic parameters (Table 2). ALT, TC, TG, and non-HDL-C decreased significantly from baseline after 6 months of oral semaglutide treatment. eGFR, AST, and HDL-C showed no significant change.

### 3.4. Safety

No patients discontinued treatment because of side effects. Among the 88 patients, nausea occurred in 10 (11.4%), with four of them being previously treated with another GLP-1RA. A headache occurred in one patient at the start of 3 mg use, but it disappeared within a few days. The doses of semaglutide at which nausea appeared were 3 mg in 5 patients, 7 mg in 3 patients, and 14 mg in 2 patients. The onset of nausea was temporary and did not persist throughout the observation period. No hypoglycemic episodes were reported.

## 4. Discussion

In this study, oral semaglutide reduced HbA1c from baseline regardless of patient background, including age, sex, and renal function, and was effective even if the previous treatment was injectable GLP-1RA. Oral semaglutide was particularly effective in lowering HbA1c with higher baseline HbA1c. Furthermore, no safety concerns were identified in the trials available in the RCTs conducted on Japanese patients.

A subgroup analysis of PIONEER 9 and 10 in Japanese patients with T2DM also suggested that higher baseline HbA1c was associated with higher HbA1c reduction, and baseline BMI or background medication was irrelevant [14]. These results were confirmed in the post hoc analysis of other PIONEER (PIONEER 3–5, 7, and 8) trials [24]. In PIONEER 9 and 10, the comparator was a pre-existing GLP-1RA, and no GLP-1RAuser was pre-treated. In this study, switching from existing GLP-1RA to oral semaglutide resulted in a significant HbA1c reduction. In PIONEER 10, dulaglutide 0.75 mg and oral semaglutide 7 mg were equally effective in lowering HbA1c [25]. In this study, the average dose of oral semaglutide was 8.52 mg, and more than 7 mg may further lower HbA1c more potently than existing GLP-1RA. It was also noted that the reduction in HbA1c tended to be slightly greater with oral semaglutide in PIONEER 9 and 10 [25,26] than in other global trials (baseline HbA1c was at similar levels), which may reflect the genetic background of diabetes and the pathogenesis of reduced insulin secretion from pancreatic beta cells but needs to be verified [27]. Switching from the existing injectable formulation to oral semaglutide may improve the patient’s treatment quality of life [9], but treatment satisfaction could not be assessed.

In the PIONEER trials (PIONEER 1–5, 7, and 8), no difference in the HbA1c-lowering effect of oral semaglutide 14 mg was observed between subgroups diabetes duration (<5 years, ≥5 to <10 years, and ≥10 years) [27], which is consistent with our results. We previously reported that once-weekly injectable semaglutide significantly reduces HbA1c from baseline regardless of disease duration, but the HbA1c-lowering effect may weaken with increasing disease duration [22]. Although this is the same RWD in patients with T2DM, the relatively short disease duration of the current patient group may have influenced the difference in results. However, PIONEER 8 was performed in patients with a longer average disease duration (about 15 years), but oral semaglutide was shown to be effective [28]. Based on the above, it is difficult to predict the effect of oral semaglutide based on the length of the disease duration. Residual pancreatic beta cell function is useful in predicting the effect of long-acting GLP-1RA [29,30]. Further studies on oral semaglutide are needed to investigate its insulin secretory capacity and HbA1c-lowering effect in detail. In this study, nausea occurred in 10 patients (11.4%), and no new side effect concerns were identified compared to existing RCTs.

Oral semaglutide was reported to significantly reduce non-fasting concentrations of triglycerides, very low-density lipoprotein, and apolipoprotein B48 (ApoB48) compared with placebo in T2DM [31]. In this study, oral semaglutide showed a −1.94% BW reduction, and weight loss was significant at 7 and 14 mg. Weight loss (kg) was not greater than that in other global studies, but this may be due to patient background (BMI). Japanese people are less likely to present with severe obesity than Westerners. However, visceral fat tends to accumulate, which, combined with decreased insulin secretion from pancreatic beta cells, leads to the development of diabetes [32,33]. Elimination of mild obesity by the Japanese is expected to improve cardiovascular metabolic markers [34]. Indeed, in a cohort study of Japanese subjects, the minimum weight loss required to improve obesity-related risk factors (such as AST, ALT, TG, and LDL-C) and conditions was reported to be 3%, and the practice is recommended [35]. However, improving obesity involves issues such as genetic background and stigma that must be overcome, making it difficult to lose weight through individual efforts alone [36,37]. In this study, oral semaglutide significantly reduced weight from baseline after 6 months, with approximately 45% of patients at 3 mg semaglutide and 54% at 7 mg achieving a 3% weight loss. In addition to lowering HbA1c, oral semaglutide may reduce BW and improve cardiovascular parameters in Japanese patients with T2DM. Liver dysfunction presenting with obesity and diabetes is considered a metabolic dysfunction-associated fatty liver disease (MAFLD) and is attracting attention as a cardiovascular risk factor [38,39]. Further studies are needed to accumulate data on obesity and MAFLD and their relationship with cardiovascular outcomes. Insulin resistance causes postprandial dyslipidemia and contributes to the development of atherosclerosis (diabetic dyslipidemia) [40]. Oral administration improved postprandial atherogenic lipid parameters (TG, non-HDL-C) in this study. We consider that the relatively small changes in lipid parameters were partly attributable to the fact that the majority of patients were using dyslipidemia medications at baseline.

This study had several limitations. First, this study was a single-center retrospective observational study without a control arm. Thus, there may be some uncorrected confounding factors. Additionally, this is a small study of a small number of cases, and the results may not be applicable to all treatments for type 2 diabetes in Japanese patients. Some safety information, such as gastrointestinal symptoms, may have been underestimated, as some of this information may not have been included in the medical records. Second, in this study, the observation period was short (6 months) to exclude the addition of other therapeutic agents as much as possible. The semaglutide dose adjustment may have been inadequate, and its effect on diabetic complications could not be studied. Third, the detail of an individual’s diet, exercise, and other habits could not be ascertained, and their effects on BW and blood glucose levels could not be examined. Fourth, fasting blood collection data were scarce and the effects of oral semaglutide on fasting blood glucose and lipid parameters could not be examined. Further evaluation under uniform blood collection conditions is needed.

## 5. Conclusions

The study results showed that oral semaglutide reduced HbA1c and BW in Japanese patients with T2DM who had failed to achieve target reductions in HbA1c with existing therapy. The HbA1c-lowering effect was found regardless of patient background, particularly with higher baseline HbA1c. These results showed that oral semaglutide may be useful for intensifying T2DM treatment in real clinical practice in Japan.

## Figures and Tables

**Table 1:** Baseline demographic and clinical characteristics.

Baseline Characteristics (*n* = 88)	
Men/women (*n*)	55/33
Age (years)	62 (53.8–68)
BMI (kg/m^2^)	27.3 (0.61)
Baseline BW (kg)	73.6 (1.58)
Diabetes duration (years)	10.5 (5–18)
Baseline HbA1c (%)	8.53 (0.17)
eGFR (mL/min/1.73 m^2^)	72.5 (50.6–95.3)
Complications	*n* (%)
Ischemic heart disease, yes, *n* (%)	17 (19.3)
Diabetic retinopathy, yes, *n* (%)	9 (10.2)
Diabetic nephropathy, yes, *n* (%)	30 (34.1)
Diabetic neuropathy, yes, *n* (%)	9 (10.2)
Anti-diabetic drugs	*n* (%)
Sulfonylurea, *n* (%)	10 (11.4)
Biguanides, *n* (%)	39 (44.3)
Glinides, *n* (%)	11 (12.5)
α-Glucosidase inhibitor, *n* (%)	13 (14.8)
Thiazolidinedione, *n* (%)	5 (5.7)
SGLT2 inhibitor, *n* (%)	60 (68.2)
Imeglimin, *n* (%)	2 (2.3)
DPP-4 inhibitor, *n* (%)	41 (46.6)
OAD only, *n* (%)	48 (54.5)
Insulin therapy, *n* (%)	23 (26.1)
Basal insulin only, *n* (%)	11 (12.5)
Bolus insulin only, *n* (%)	1 (1.1)
IDegAsp, *n* (%)	3 (3.4)
Basal-Bolus therapy, *n* (%)	8 (9.1)
GLP-1RA, *n* (%)	31 (35.2)
Liraglutide, *n* (%)	3 (3.4)
Lixisenatide, *n* (%)	3 (3.4)
Dulaglutide, *n* (%)	17 (19.3)
Once-weekly semaglutide, *n* (%)	8 (9.1)
Other drugs	
Statin use, yes, *n* (%)	52 (59.1)
Fibrate use, yes, *n* (%)	4 (4.5)
Ezetimibe use, yes, *n* (%)	10 (11.4)
Anti-hypertensive drug, yes, *n* (%)	48 (54.5)

Data are presented as numbers (%) for categorical variables and as means (±standard error of the mean, SEM) or median with interquartile range (IQR) for continuous variables. BW, body weight; BMI, body mass index; HbA1c, glycated hemoglobin; eGFR, estimated glomerular filtration rate; SGLT2, sodium-glucose cotransporter 2; DPP-4, dipeptidyl peptidase-4; OAD, oral anti-diabetic drug; GLP-1RA, glucagon-like peptide-1 receptor agonist.

**Table 2:** Changes in cardiometabolic parameters after 6 months of oral semaglutide treatment.

Clinical Parameters	*n*	Baseline	After 6 Months	*p* Value
AST (IU/L)	88	22 (18–30.5)	23 (19.5–28)	0.065
ALT (IU/L)	88	24 (17.8–38.3)	23 (20–32)	0.008
eGFR (mL/min/1.73 m^2^)	88	74.5 (3.2)	72.6 (3.3)	0.055
TC (mg/dL)	72	177 (4.5)	172 (4.2)	0.009
HDL-C (mg/dL)	86	49.5 (40.3–56)	49 (41–57.8)	0.462
TG (mg/dL)	87	146 (110–202)	144 (114–181)	0.028
Non-HDL-C (mg/dL)	71	120 (98–158)	115 (98–149)	0.002

Data are presented as means (±standard error of the mean, SEM) or median with interquartile range (IQR). *p* values are calculated by the Wilcoxon signed-rank sum test (vs. baseline parameters). AST, aspartate aminotransferase; ALT, alanine aminotransferase; eGFR, estimated glomerular filtration rate; TC, total cholesterol; HDL-C, high-density lipoprotein cholesterol; TG, triglyceride.

## Data Availability

All data generated or analyzed during this study are included in this article, along with references to data from cited published studies. The database is not publicly available. Further inquiries can be directed to the corresponding author.

## Supplementary Materials

The following supporting information can be downloaded at: https://www.mdpi.com/article/10.3390/jcdd10040176/s1, Figure S1: Change in HbA1c according to baseline BMI (A) and diabetes duration (B); Figure S2: Change in HbA1c according to baseline HbA1c category; Figure S3: Change in HbA1c and BW from baseline in prior DPP-4 inhibitor (user or, not); Table S1: Effect of baseline clinical parameters on the good response group.
