# Supplementary material for: Retrospective Analysis of the Effectiveness of Oral Semaglutide in Type 2 Diabetes Mellitus and Its Effect on Cardiometabolic Parameters in Japanese Clinical Settings
Source: J Cardiovasc Dev Dis. 2023 Apr 18;10(4):176. doi: 10.3390/jcdd10040176 (PMC10141082; doi:10.3390/jcdd10040176)
Supplement: Supplementary file 1 [file jcdd-10-00176-s001.zip › jcdd-2321098-supplementary.pdf]

Supplementary Materials

Retrospective Analysis of the Effectiveness of Oral Semaglutide in Type 2 Diabetes Mellitus and Its Effect on Cardiometabolic Parameters in Japanese Clinical Settings

Hodaka Yamada [1], Masashi Yoshida [1], Shunsuke Funazaki [1], Jun Morimoto [1], Shiori Tonezawa [1], Asuka Takahashi [1], Shuichi Nagashima [1], Kimura Masahiko [2], Otsuka Kiyoshi [2] and Kazuo Hara [1]

[1] Department of Medicine, Division of Endocrinology and Metabolism, Jichi Medical University Saitama Medical Center, 1-847 Amanuma-cho, Omiya-ku, Saitama 330-8503, Japan

[2] Department of Pharmacy, Jichi Medical University Saitama Medical Center, 1-847 Amanuma-cho, Omiya-ku, Saitama 330-8503, Japan

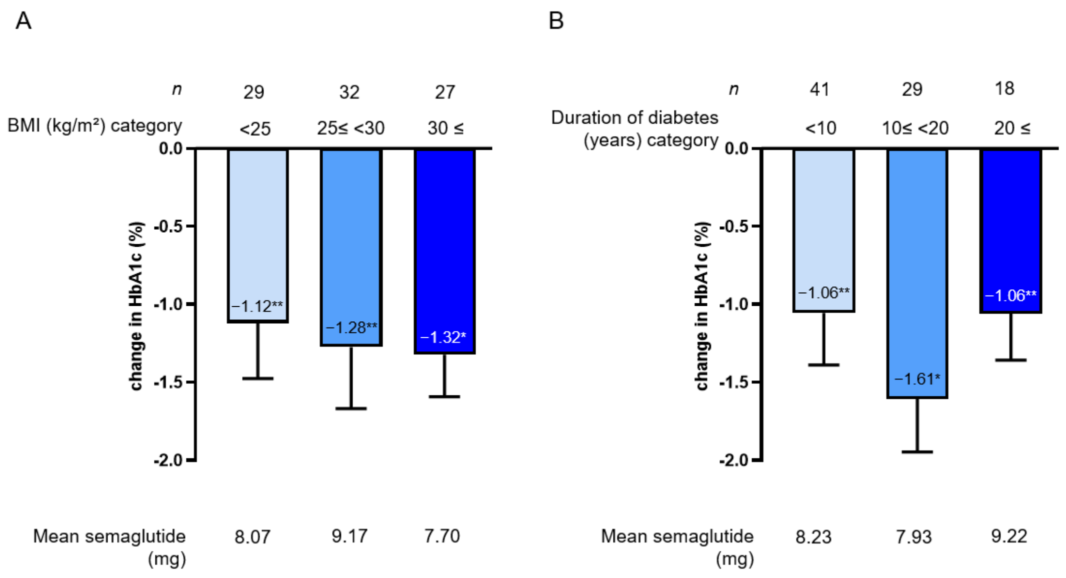

Figure S1. Change in HbA1c according to baseline BMI (**A**) and diabetes duration (**B**). The mean oral semaglutide dosage is shown at the bottom of the figure. Data in the figure are presented as mean with SEM. * $p < 0.001$; ** $p < 0.01$ in the Wilcoxon signed-rank sum test (vs. BL HbA1c). BL, baseline; HbA1c, glycated hemoglobin; BMI, body mass index; SEM, standard error of the mean.

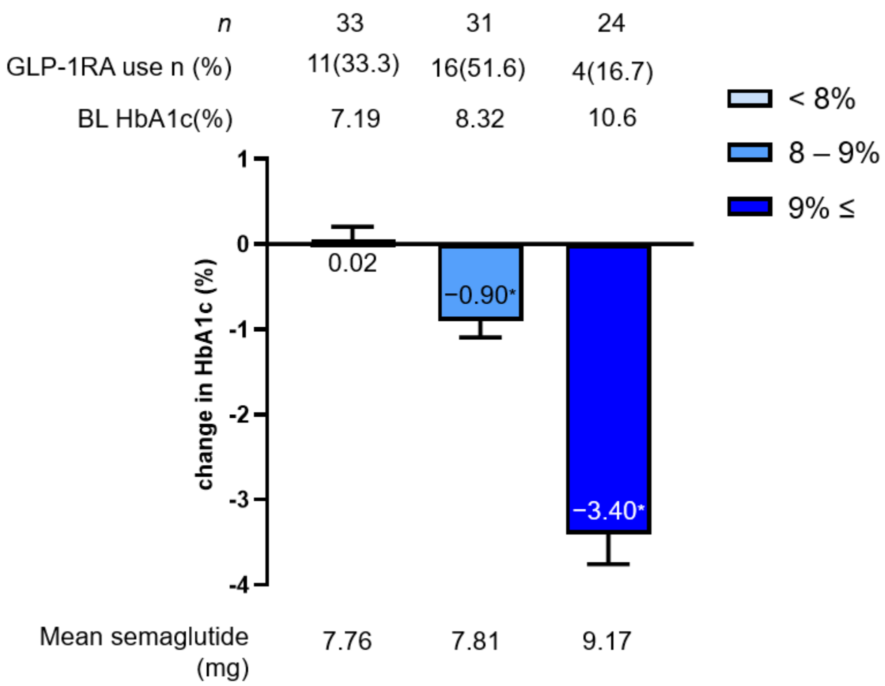

Figure S2. Change in HbA1c according to baseline HbA1c category. The mean oral semaglutide dosage is shown at the bottom of the figure. Data in the figure are presented as mean with SEM. * $p < 0.001$ in the Wilcoxon signed-rank sum test (vs. BL HbA1c). BL, baseline; HbA1c, glycated hemoglobin; BMI, body mass index; GLP-1RA, glucagon-like peptide-1 receptor agonist; SEM, standard error of the mean.

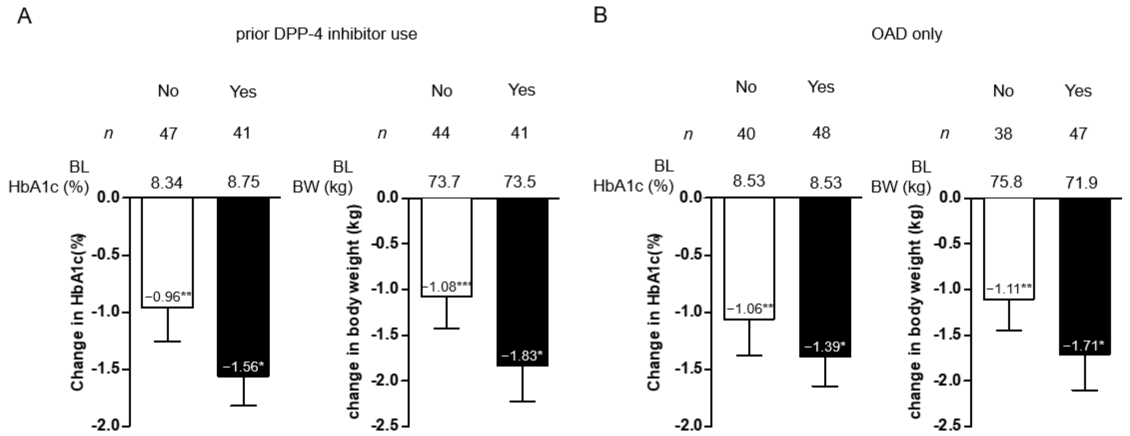

Figure S3. Change in HbA1c and BW from baseline in prior DPP-4 inhibitor (user or, not) (**A**) and OAD usage (OAD only or, not) (**B**). Data in the figure are presented as mean with SEM. * $p <$ 0.001; ** $p < 0.01$; *** $p < 0.05$ in the Wilcoxon signed-rank sum test (vs. BL HbA1c). BL, baseline; HbA1c, glycated hemoglobin; DPP-4, Dipeptidyl peptidase-4; OAD, oral anti-diabetic drug; SEM, standard error of the mean.

Table S1. Effect of baseline clinical parameters on the good-response group.

**Clinical Parameters**	**OR**	**95%CI**	***p* Value**
Age (≥65)	0.982	0.34–2.84	0.974
Gender (women)	1.37	0.464–4.05	0.569
BMI (≥25)	0.542	0.17–1.73	0.302
Diabetes duration (≥10)	1.66	0.573–4.83	0.349
eGFR (≦60)	1.46	0.516–4.15	0.474
Baseline HbA1c (≥8.5)	8.45	2.96–24.1	<0.001

BMI, body mass index; eGFR, estimated glomerular filtration rate; HbA1c, glycated hemoglobin; OR, odds ratio; CI, confidence interval.
